# Self-Reported Weight Gain After the Age of 20 and Risk of Steatotic Liver Disease

**DOI:** 10.3390/nu17152566

**Published:** 2025-08-06

**Authors:** Masayo Iwasa, Naoki Ozu, Hajime Yamakage, Hisashi Kato, Misato Ishikawa, Megumi Kanasaki, Izuru Masuda, Masashi Tanaka, Noriko Satoh-Asahara

**Affiliations:** 1 Department of Endocrinology, Metabolism and Hypertension Research, Clinical Research Institute, NHO Kyoto Medical Center, 1-1 Fukakusa Mukaihata-cho, Fushimi-ku, Kyoto 612-8555, Japan; 2 Institute for Clinical and Translational Science, Nara Medical University Hospital, Kashihara 634-8522, Japan; 3 Department of Metabolic Research, Geroscience Research Center, Research Institute, National Center for Geriatrics and Gerontology, 7-430 Morioka-cho, Obu 474-8511, Japan; 4 Takeda Hospital Medical Checkup Center, Nihon Seimei Kyoto Santetsu Building, 608, Higashishiochoji-cho, Nishinotoin Higashi-iru, Shiokoji-dori, Shimogyo-ku, Kyoto 600-8216, Japan; 5 Department of Diabetes Mellitus, Mitsubishi Kyoto Hospital, 1 Katsuragosho-cho, Nishikyo-ku, Kyoto 615-8087, Japan; 6 Department of Rehabilitation, Health Science University, 7187 Kodachi, Fujikawaguchiko-machi, Minamitsuru-gun 401-0380, Japan; 7 Department of Diabetes and Endocrinology, Hospital, National Center for Geriatrics and Gerontology, 7-430 Morioka-cho, Obu 474-8511, Japan; 8 Department of Clinical Laboratory, Hospital, National Center for Geriatrics and Gerontology, 7-430 Morioka-cho, Obu 474-8511, Japan

**Keywords:** a longitudinal study, body mass index, hepatic steatosis, questionnaire items, the general population, universal screening, weight gain history

## Abstract

**Background/Objectives**: We aimed to identify questionnaire items associated with an increased risk of developing hepatic steatosis in the general population. **Methods**: A total of 15,063 individuals aged ≥20 years who underwent general health checkups and had no hepatic steatosis at baseline were included. The relationship between questionnaire data at baseline and hepatic steatosis incidence over a median 4.2-year follow-up was investigated across body mass index (BMI) categories. **Results**: Among 15,063 individuals (mean [SD] age, 47.1 [10.2] years; 6769 [44.9%] male; mean [SD] BMI, 21.4 [2.6] kg/m^2^), 1889 individuals (12.5%) developed hepatic steatosis during follow-up. After adjusting for age, sex, and factors related to metabolic diseases and liver injury, the strongest questionnaire-based risk factor for hepatic steatosis was self-reported weight gain of 10 kg or more after the age of 20 across all BMI categories: total population (hazard ratio [HR], 2.11; 95% confidence interval [CI], 1.90–2.34; *p* < 0.001), Category 1 (BMI < 22) (HR, 2.33; 95% CI, 1.86–2.91; *p* < 0.001), Category 2 (BMI 22 to <25) (HR, 1.43; 95% CI, 1.25–1.63; *p* < 0.001), and Category 3 (BMI ≥ 25) (HR, 1.41; 95% CI, 1.12–1.77; *p* = 0.003). **Conclusions**: In this cohort study, self-reported weight gain of 10 kg or more after the age of 20 was associated with an increased risk of hepatic steatosis, independent of baseline BMI. Questionnaires capturing weight gain history may support universal screening efforts to identify individuals at elevated risk.

## 1. Introduction

Steatotic liver disease (SLD) is a global health concern associated with high mortality rates, affecting approximately 30% of the global population [1,2,3,4]. SLD is linked to an increased risk of cardiovascular disease (CVD), metabolic diseases, and chronic kidney disease [2]. There is an urgent need to develop effective strategies for preventing and managing SLD.

SLD refers to hepatic steatosis of various etiologies and is classified into categories based on cardiometabolic risk factors, alcohol consumption, or other defined causes: metabolic dysfunction-associated SLD, metabolic and alcohol-associated liver disease, alcohol-associated liver disease, SLD of other specific etiologies, and cryptogenic SLD [5]. Each category presents distinct clinical features and is associated with different risks for comorbidities. However, a shared pathological hallmark is ectopic lipid accumulation in the liver. Notably, this process is preventable and reversible with appropriate intervention [6]. These findings underscore the value of early detection in preventing disease progression, regardless of the underlying etiology.

Although numerous studies have explored methods for assessing hepatic steatosis, universal SLD screening remains challenging. While obesity is a well-established contributor to hepatic fat deposition, hepatic steatosis also occurs in individuals with normal weight [7,8]. Therefore, body mass index (BMI) alone may not adequately estimate hepatic steatosis, even though fat accumulation from weight gain is involved in SLD pathogenesis. Blood-based biomarkers have shown some utility, but their accuracy can be influenced by confounding variables [9]. Imaging techniques, such as abdominal ultrasonography and magnetic resonance imaging, are effective but resource dependent [10]. Thus, hepatic steatosis indicators that do not require specialized equipment and are suitable for population-level screening are needed.

Accumulating evidence reveals that lifestyle factors such as weight gain, physical inactivity, and unhealthy diet are associated with the risk of hepatic steatosis [7]. By addressing these factors, SLD can be prevented, and making lifestyle improvements is currently the mainstay for SLD management [11]. Therefore, lifestyle factors can be considered as risk factors in the universal screening of SLD. However, the factors have not been fully elucidated.

Questionnaires are among the most widely used tools in clinical assessments and offer a practical means for gathering lifestyle, dietary, and symptom-related information from individuals. They also allow for the collection of self-reported data on changes in body composition. Identifying questionnaire items associated with hepatic steatosis could support early recognition of high-risk individuals across all SLD categories. This may facilitate timely intervention and risk communication through same-day, questionnaire-based feedback. In this study, we investigated the potential utility of questionnaires in identifying individuals at increased risk of hepatic steatosis by longitudinally analyzing lifestyle information collected from a general population health examination database.

## 2. Materials and Methods

### 2.1. Participants

This study was designed to investigate the longitudinal relationship between baseline questionnaire data and the onset of hepatic steatosis over a 5-year follow-up period in the general Japanese population. Altogether, 18,384 individuals underwent general health checkups at the Takeda Hospital Medical Examination Center from 2011 to 2015. These individuals further underwent abdominal ultrasonography, an optional medical examination, and had no hepatic steatosis at baseline, followed by at least one ultrasonographic evaluation during the follow-up period. Of these, 3321 individuals were excluded because of ongoing treatment for diabetes, hypertension, or dyslipidemia or a history of liver disease, CVD, or cerebrovascular disease to prevent the potential effect of therapies (e.g., medications) or anamnesis on the outcomes. Ultimately, 15,063 individuals were included in this study. The study was approved by the institutional review board (Medical Examination Center, Takeda Hospital, “22-11”) and conducted in accordance with the Declaration of Helsinki and the Ethical Guidelines for Medical and Health Research Involving Human Subjects. Informed consent was obtained through an opt-out method by posting an explanation at the Takeda Hospital Medical Examination Center.

### 2.2. Anthropometric and Metabolic Parameters

The following measurements were conducted at baseline and follow-up. BMI was calculated as weight (kg) divided by height squared (m^2^). Systolic blood pressure (SBP) and diastolic blood pressure (DBP) were also measured. Venous blood samples were collected after a 12 h fasting period to determine the fasting plasma glucose (FPG), hemoglobin A1c (HbA1c), total cholesterol, triglycerides (TGs), high-density lipoprotein cholesterol (HDL-C), low-density lipoprotein cholesterol (LDL-C), aspartate aminotransferase (AST), alanine aminotransferase (ALT), and γ-glutamyltransferase (γ-GT) levels, using standard laboratory techniques [12].

### 2.3. Questionnaire Items on Eating Habits and Lifestyle Behaviors

The questionnaire used in this study was developed according to the standardized instrument provided by the Japanese Ministry of Health, Labour and Welfare for the Specific Health Checkup Program. It consists of 23 items, covering (1) eating habits and behaviors, (2) smoking and drinking habits, (3) exercise habits, (4) weight fluctuation, and (5) sleep. Response options included “yes,” “no,” and multiple-choice answers such as “every day,” “sometimes,” or “rarely,” as outlined in Table 1. Questionnaire data obtained at baseline were investigated to determine whether they were associated with the incidence of hepatic steatosis.

### 2.4. Abdominal Ultrasonography for Detection of Hepatic Steatosis

Abdominal ultrasonography was performed at baseline and follow-up to detect hepatic steatosis, using the Xario100 and Xario200 diagnostic imaging systems (Canon Medical Systems Corporation, Otawara, Japan) equipped with a 3.5 MHz convex array transducer. Images of the hepatic parenchyma were used to assess liver echogenicity. All examinations were performed by sonographers with at least 5 years of experience who were trained by gastroenterologists with more than 5 years of experience. Each certified gastroenterologist independently reviewed the images and evaluated the liver for the presence of hepatic steatosis.

### 2.5. Statistical Analysis

The Japan Society for the Study of Obesity defines obesity as a BMI ≥ 25, based on the World Health Organization criteria for overweight and evidence that a BMI ≥ 25 increases obesity-related complications in Japanese individuals [13,14]. In addition, a BMI of 22 is considered the ideal BMI for the Japanese population [15]. Therefore, in this study, the participants were categorized into three groups based on baseline BMI: Category 1 (BMI < 22), Category 2 (BMI 22 to <25), and Category 3 (BMI ≥ 25). The goodness-of-fit test was conducted to determine whether there was a bias in the distribution of the number of individuals across the BMI categories.

Questionnaire items with 3 or more response options were dichotomized, as shown in Table 1. For each question, the reference was as follows: Question No. 1–5, 7, 9, 11, and 12, “No”; Question No. 6, “Normal” or “Slow”; Question No. 8, 10, and 14–23, “Sometimes” or “Rarely”; and Question No. 13, “Eating until 80% full” or “No.” Continuous background variables were presented as mean (standard deviation [SD]) and categorical variables as number (percent). Group differences were evaluated using the chi-square test with Bonferroni correction for categorical variables and the Tukey–Kramer test for continuous variables to identify significant pairwise differences among the groups. The incidence of hepatic steatosis across BMI categories was compared by log-rank test for trend.

The Cox proportional hazards model was used to estimate the adjusted hazard ratios (HRs) and 95% confidence intervals (CIs) for the association between baseline questionnaire data and the incidence of hepatic steatosis over the 5-year follow-up period. HRs were first adjusted for age and sex (Model 1). Model 2 included additional adjustments for metabolic factors: impaired glucose metabolism (FPG ≥ 100 mg/dL and/or HbA1c ≥ 5.6%), hypertension (SBP ≥ 140 mmHg and/or DBP ≥ 90 mmHg), dyslipidemia (LDL-C ≥ 140 mg/dL and/or TG ≥ 150 mg/dL and/or HDL-C < 40 mg/dL), and liver injury (ALT ≥ 31 U/L).

A two-sided alpha was set at 0.05. All statistical analyses were conducted using SPSS version 24.0 for Windows (SPSS Inc., Chicago, IL, USA).

## 3. Results

### 3.1. Baseline Characteristics

The participants’ baseline characteristics are summarized in Table 2. A total of 15,063 individuals were included in this study (6769 males [44.9%] and 8294 females [55.1%]), with a mean age of 47.1 years and a mean BMI of 21.4 kg/m^2^. The distribution of individuals across BMI categories was as follows: 9270 in Category 1, 4519 in Category 2, and 1274 in Category 3. The goodness-of-fit test revealed a statistically significant deviation from uniform distribution. Notably, the number of individuals in Category 1 was disproportionately high (61.5%), whereas Category 3 had a relatively small proportion (8.5%).

Blood pressure and glucose metabolism-related indices were higher in the high BMI categories than in the low BMI categories (Category 1 vs. Category 2, Category 1 vs. Category 3, and Category 2 vs. Category 3) (Table 2). Liver function-related parameters were also exacerbated in higher BMI categories, except for total cholesterol (Category 2 vs. Category 3, *p* = 0.738) and LDL-C (Category 2 vs. Category 3, *p* = 0.056).

### 3.2. Relationship Between Questionnaire Items and the Development of Hepatic Steatosis (Model 1)

The participants’ characteristics at the end of the follow-up period are summarized in Table 3. Blood pressure, glucose metabolism-related indices, and liver function-related parameters remained exacerbated in higher BMI categories than in lower BMI categories as observed at baseline. The follow-up period was shorter in the higher BMI categories. The changes in mean body weight between the baseline and the end of the follow-up period were less than ±1 kg in all the BMI categories.

Among the 15,063 participants, 1889 individuals (12.5%) developed hepatic steatosis during the follow-up period (median follow-up, 4.2 years; maximum, 5.5 years) (Table 3). Incident hepatic steatosis was observed in 551 individuals in Category 1 (5.9% of 9270 participants), 898 in Category 2 (19.9%), and 440 in Category 3 (34.5%). The incidence of hepatic steatosis increased significantly with increasing BMI (*p* for trend < 0.001). Therefore, we examined whether questionnaire items were associated with the risk of developing hepatic steatosis in the overall population as well as within each baseline BMI category using Model 1, which was adjusted for age and sex.

**Figure 1:**
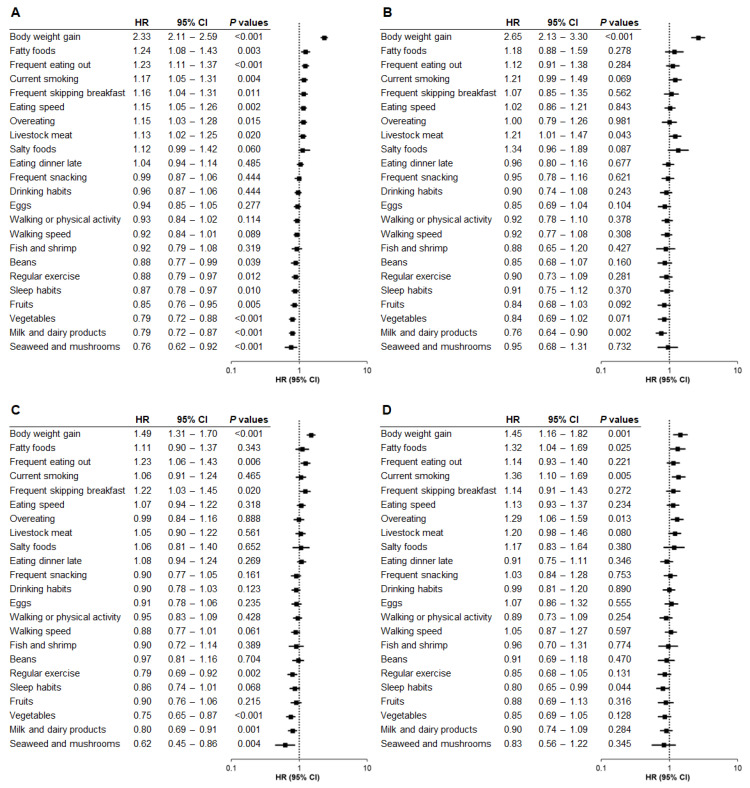
Relationship between questionnaire items and hepatic steatosis by baseline BMI category (Model 1). The association between questionnaire items and the risk of developing hepatic steatosis was analyzed based on baseline body mass index (BMI) category using Model 1, adjusted for age and sex. (**A**) Total population. (**B**) BMI < 22. (**C**) BMI 22 to <25. (**D**) BMI ≥ 25. HR, hazard ratio; CI, confidence interval.

In the total population, the strongest questionnaire-based risk factor for hepatic steatosis was self-reported weight gain of 10 kg or more after the age of 20 (HR, 2.33; *p* < 0.001) (Figure 1A). Conversely, the lowest risk factor was daily consumption of seaweed and mushrooms (HR, 0.76; *p* = 0.006).

Next, we explored the relationship between questionnaire data and hepatic steatosis onset across BMI categories. Notably, self-reported weight gain of 10 kg or more after the age of 20 was the strongest risk factor in Category 1 (HR, 2.65; *p* < 0.001) (Figure 1B), indicating that self-reported weight gain at baseline substantially increased the risk of hepatic steatosis within 5 years, despite the baseline BMI being <22. The same weight gain item was also the highest risk factor in Category 2 (HR, 1.49; *p* < 0.001) (Figure 1C) and Category 3 (HR, 1.45; *p* = 0.001) (Figure 1D). These findings suggest that self-reported weight gain of 10 kg or more after the age of 20 was the strongest risk factor for hepatic steatosis across all BMI categories. Moreover, the associated risk was higher in Category 1 than in Category 2 (*p* < 0.001) or Category 3 (*p* < 0.001), with no significant difference between Categories 2 and 3 (*p* = 0.840).

No questionnaire item was commonly associated with either increased or decreased risk of hepatic steatosis across all BMI categories except for self-reported weight gain. However, the following questionnaire items at baseline were most strongly associated with a reduced risk of hepatic steatosis in each BMI group: daily consumption of milk and dairy products in Category 1 (HR, 0.76; *p* = 0.002) (Figure 1B), daily consumption of seaweed and mushrooms in Category 2 (HR, 0.62; *p* = 0.004) (Figure 1C), and self-reported sleep satisfaction in Category 3 (HR, 0.80; *p* = 0.044) (Figure 1D).

Our analyses revealed additional questionnaire items that were associated with the risk of hepatic steatosis incidence in each BMI category. For Category 1, daily intake of livestock meat was a significant risk factor (HR, 1.21; *p* = 0.043) (Figure 1B). For Category 2, frequent eating out (HR, 1.23; *p* = 0.006) and frequent skipping breakfast (HR, 1.22; *p* = 0.020) were associated with an elevated risk of hepatic steatosis, while regular exercise (HR, 0.79; *p* = 0.002), daily consumption of vegetables (HR, 0.75; *p* < 0.001), and daily intake of milk and dairy products (HR, 0.80; *p* = 0.001) were associated with a reduced risk (Figure 1C). For Category 3, daily consumption of fatty foods (HR, 1.32; *p* = 0.025), current smoking (HR, 1.36; *p* = 0.005), and overeating (HR, 1.29; *p* = 0.013) were significantly associated with the risk of hepatic steatosis (Figure 1D), while no questionnaire item, including regular exercise, was significantly associated with the decreased risk of hepatic steatosis (Figure 1D).

### 3.3. Relationship Between Questionnaire Items and the Development of Hepatic Steatosis (Model 2)

**Figure 2:**
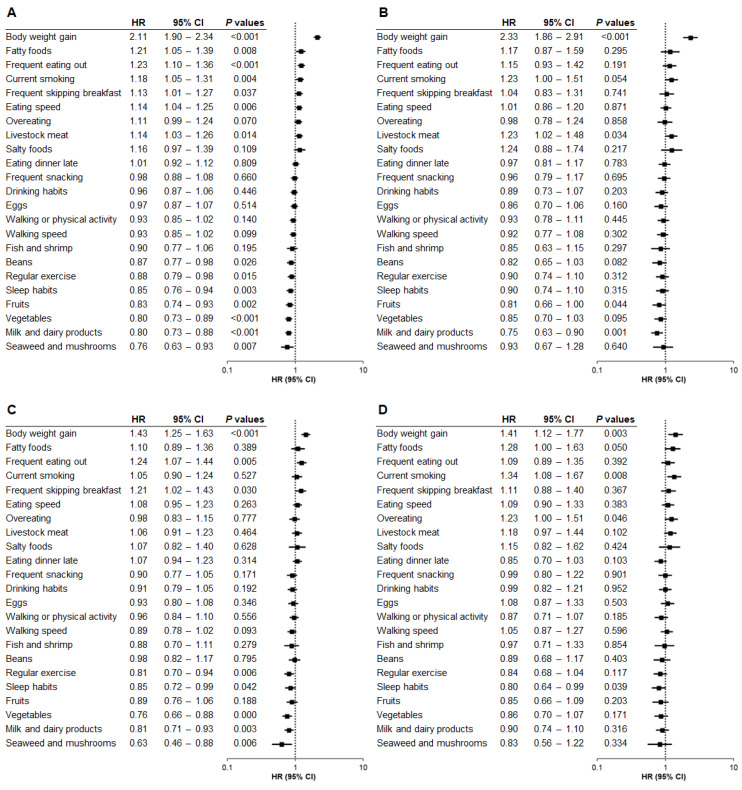
Relationship between questionnaire items and hepatic steatosis by baseline BMI category (Model 2). The association between questionnaire items and the risk of developing hepatic steatosis was analyzed by baseline body mass index (BMI) category using Model 2, adjusted for age, sex, and factors related to metabolic diseases and liver injury. (**A**) Total population. (**B**) BMI < 22. (**C**) BMI 22 to <25. (**D**) BMI ≥ 25. HR, hazard ratio; CI, confidence interval.

We further examined the relationship between baseline questionnaire data and the onset of hepatic steatosis using Model 2, which was adjusted for age, sex, and factors related to metabolic diseases and liver injury. Self-reported weight gain of 10 kg or more after the age of 20 remained the strongest risk factor for hepatic steatosis across all subgroups: total population (HR, 2.11; *p* < 0.001) (Figure 2A), Category 1 (HR, 2.33; *p* < 0.001) (Figure 2B), Category 2 (HR, 1.43; *p* < 0.001) (Figure 2C), and Category 3 (HR, 1.41; *p* = 0.003) (Figure 2D). Additionally, the risk associated with this weight gain episode was significantly higher in Category 1 than in Category 2 (*p* < 0.001) or Category 3 (*p* < 0.001), while no significant difference was observed between Category 2 and Category 3 (*p* = 0.694).

Although no common protective questionnaire item was identified across all BMI categories apart from weight gain, the following items were most strongly associated with a reduced risk of hepatic steatosis: daily consumption of milk and dairy products in Category 1 (HR, 0.75; *p* = 0.001) (Figure 2B), daily consumption of seaweed and mushrooms in Category 2 (HR, 0.63; *p* = 0.006) (Figure 2C), and self-reported sleep satisfaction in Category 3 (HR, 0.80; *p* = 0.039) (Figure 2D).

Other questionnaire items associated with the risk of hepatic steatosis in Category 1 were daily consumption of livestock meat (HR, 1.23; *p* = 0.034) and fruits (HR, 0.81; *p* = 0.044) (Figure 2B). For Category 2, frequent eating out (HR, 1.24; *p* = 0.005) and frequent skipping breakfast (HR, 1.21; *p* = 0.030) were associated with an increased risk, but regular exercise (HR, 0.81; *p* = 0.006), self-reported sleep satisfaction (HR, 0.85; *p* = 0.042), daily intake of vegetables (HR, 0.76; *p* < 0.001), and daily consumption of milk and dairy products (HR, 0.81; *p* = 0.003) were associated with a decreased risk (Figure 2C). For Category 3, current smoking (HR, 1.34; *p* = 0.008) and overeating (HR, 1.23; *p* = 0.046) were significantly associated with the risk of hepatic steatosis (Figure 2D).

## 4. Discussion

This is the first study to provide evidence that self-reported weight gain of 10 kg or more after the age of 20 is a novel risk factor for the development of hepatic steatosis within 5 years, regardless of the baseline BMI category. We also identified questionnaire items associated with a decreased risk of hepatic steatosis for each BMI category: daily consumption of milk and dairy products, daily consumption of seaweed and mushrooms, and sleep satisfaction. These findings support the utility of questionnaire-based “same-day feedback,” potentially increasing awareness and encouraging lifestyle modifications among the respondents that contribute to the prevention of hepatic steatosis.

Our findings indicate that the strongest baseline risk factor for hepatic steatosis was self-reported weight gain of 10 kg or more after the age of 20 across all BMI categories after adjusting for age, sex, and metabolic factors including blood markers. Given that hepatic steatosis can develop in individuals without obesity as well as in those with obesity [7,8], this result suggests that hepatic steatosis in individuals without obesity may be partially attributable to prior weight gain. These results underscore the value of incorporating weight gain history into risk screening tools, as such information is inherently personal and cannot be captured by diagnostic equipment alone. Therefore, this weight gain item may serve as a practical and effective screening question for identifying individuals at high risk of hepatic steatosis, irrespective of BMI category.

We acknowledge the distributional heterogeneity across BMI categories in this study; the number of participants in each BMI category decreased with increasing BMI. This may reflect the distributional characteristics of individuals with no hepatic steatosis in the general population because this study excluded those with hepatic steatosis at baseline. Nevertheless, self-reported weight gain of 10 kg or more after the age of 20 was the strongest risk factor, independent of baseline BMI. The associated risk also remained significant even after adjusting for confounding factors. Furthermore, the changes in mean body weight were not substantial during the follow-up period in each BMI group and comparable across BMI categories, suggesting the unlikelihood of higher rates of body weight gain being associated with the incidence of hepatic steatosis in higher BMI categories in this study. These findings corroborate the notion that the self-reported weight gain history is useful for identifying individuals at elevated risk of hepatic steatosis, regardless of age, BMI, or metabolic disease-related indices at the baseline.

Our findings provide novel insights into the potential utility of questionnaires in hepatic steatosis prevention. Once high-risk individuals are identified, questionnaires can guide lifestyle interventions tailored to baseline BMI—for example, daily consumption of milk and dairy products, daily consumption of seaweed and mushrooms, and satisfaction with sleep quality. Accordingly, questionnaires may serve dual functions: risk screening based on weight gain episodes and risk reduction through BMI-specific lifestyle recommendations. These questionnaire-based strategies are concise, feasible, and can be implemented on the same day as the health examinations, enabling timely feedback to participants. Furthermore, because hepatic steatosis is a central pathological feature of SLD with diverse etiologies, reducing its risk may contribute to the prevention of multiple liver-related conditions. We, therefore, advocate for the inclusion of weight gain history in standard questionnaires as a universal and practical tool for screening high-risk individuals.

Interestingly, when weight gain of 10 kg or more after the age of 20 was reported, individuals with baseline BMI < 22 had a higher risk of hepatic steatosis than those with BMI ≥ 22. Although the underlying mechanisms remain unclear, adipose tissue lipid storage capacity may play a role in this association [16]. The number of adipocytes, a key determinant of fat mass in adults, is established during childhood and adolescence and remains stable in adulthood [17], suggesting that BMI prior to adulthood may influence lipid storage capacity later in life [16]. Therefore, the amount of lipids by weight gain during adulthood would exceed the storage capacity of adipose tissue in individuals with a low BMI before adulthood, and weight gain in adulthood may exceed the storage threshold of adipose tissue [16,17], leading to excessive ectopic lipid accumulation [16]. This may explain the higher hepatic steatosis risk observed in participants with baseline BMI < 22. Individuals with low BMI may underestimate their risk for hepatic steatosis; however, our findings highlight the importance of recognizing weight gain in adulthood as a potential risk indicator, even in the absence of obesity.

Consumption of milk or dairy products has been reported to be associated with a reduced risk of non-alcoholic fatty liver disease (NAFLD), possibly due to dairy components that protect and improve liver function [18,19]. Seaweeds and mushrooms also contain bioactive compounds with antioxidative and anti-inflammatory properties [20,21,22]. The intake of seaweeds or their components beneficially influenced NAFLD [23,24] and newly diagnosed NAFLD [22]. The potential of mushrooms in preventing or improving NAFLD has been reported [25], and their intake has been inversely associated with the prevalence of newly diagnosed NAFLD [26]. Improving sleep quality has similarly been linked to a reduced risk of metabolic dysfunction-associated fatty liver disease (MAFLD) [27,28], highlighting the role of sleep as one of several traditional low-risk lifestyle factors (e.g., a healthy diet) [28]. These findings support our results that daily consumption of milk and dairy products, daily consumption of seaweed and mushrooms, and sleep satisfaction, assessed via questionnaires, were negatively associated with the risk of hepatic steatosis. Furthermore, because BMI may influence hepatic steatosis risk factors such as glucose and lipid metabolism, the most appropriate lifestyle interventions may vary by BMI category, which could explain the differences in low-risk questionnaire items observed among the BMI groups.

Although previous studies reported beneficial effects of exercise on NAFLD [29,30] and MAFLD [31,32], the preventive effects were limited to participants with baseline BMI 22 to <25, with no significant effects on those with BMI < 22 or BMI ≥ 25, in this study. Given that obesity contributes to hepatic lipid accumulation and participants in this study had no hepatic steatosis at baseline, the risk of fat deposition in the liver remained low in individuals with low BMI if they do not have weight gain history, in whom the beneficial effects of exercise might not be pronounced. When BMI increases, the risk of hepatic fat deposition increases accordingly, and this could exacerbate the effects of hepatic steatosis-related factors and reduce the effects of exercise. These characteristics of individuals with low or high baseline BMI in the general population might limit the effects of exercise on preventing hepatic steatosis incidence. Future studies are needed to elucidate the mechanisms underlying the relationship between effects of exercise and BMI category. Nevertheless, our findings suggest that exercise can be offered to individuals with a baseline BMI of 22 to <25 as a protective factor against hepatic steatosis.

This study has some limitations. First, questionnaire data were collected through self-reporting, which may have introduced reporter or recall bias. Nonetheless, the questionnaire items used in this study were derived from the standardized questionnaire developed by the Japanese Ministry of Health, Labour and Welfare, which is validated and widely used [33,34]. Second, hepatic steatosis was diagnosed using ultrasonography rather than histological evaluation. However, liver biopsy is not appropriate for population-based epidemiological studies [35], and ultrasonography is a recommended, widely accepted, and non-invasive diagnostic method for hepatic steatosis [28,36]. Third, the questionnaire captured lifestyle data only at baseline; the participants may have modified their behaviors, such as dietary and exercise habits, during the follow-up period. Future studies should track these lifestyle changes and assess their effects on hepatic steatosis risk over time. Finally, the generalizability of our findings may be limited, as the findings of this study are based on a single-center study. Furthermore, lifestyle habits differ across ethnicities and regions. Nevertheless, the large sample size enhances external validity and allows for cautious extrapolation to broader populations. Clarifying the role of questionnaire items across different racial and ethnic groups could help identify high- and low-risk patterns specific to those populations.

## 5. Conclusions

This study highlights the potential utility of questionnaires in identifying and reducing the risk of hepatic steatosis. Self-reported weight gain episodes at baseline may enable immediate identification of individuals at high risk for hepatic steatosis within 5 years, regardless of baseline BMI. Furthermore, tailored lifestyle recommendations could be promptly offered based on low-risk questionnaire items corresponding to each BMI category. This questionnaire-based “same-day feedback” approach may help reduce the risk of hepatic steatosis and contribute to the prevention of SLD, in which hepatic steatosis represents the fundamental pathology.

## Figures and Tables

**Table 1:** Questionnaire items on lifestyles.

Question No.	Category	Subcategory	Questions	Answer Choices
1	Specific Health Checkups	Current smoking	Are you currently a regular smoker?	Yes/No
2		Body weight gain	Have you gained 10 kg or more since the age of 20?	Yes/No
3		Regular exercise	Have you engaged in exercise at least 2 days per week, for a minimum of 30 min each time, at an intensity that causes slight sweating, and continued this habit for at least 1 year?	Yes/No
4		Walking or physical activity	Do you walk for at least 1 h per day or perform an equivalent level of physical activity in your daily routine?	Yes/No
5		Walking speed	Do you walk faster than most individuals of your age and sex?	Yes/No
6		Eating speed	How quickly do you eat compared to others? (e.g., fast, moderate, slow)	Fast/Normal/Slow
7		Eating dinner late	Do you eat dinner within 2 h before going to bed on 3 or more days per week?	Yes/No
8		Frequent snacking	Do you consume snacks or sweetened beverages outside of breakfast, lunch, and dinner?	Everyday/Sometimes/Rarely
9		Frequent skipping breakfast	Do you skip breakfast on 3 or more days per week?	Yes/No
10		Drinking habits	How often do you consume alcoholic beverages (e.g., sake, shochu, beer, liquor)?	Everyday/Sometimes/Rarely (cannot drink)
11		Sleep habits	Do you feel refreshed upon waking after a night’s sleep?	Yes/No
12	Eating habits questions	Frequent eating out	How often do you eat meals outside the home?	Yes/No
13		Overeating	Do you always overeat?	Yes/Eating until 80% full/No
		Dietary habits		
14		Livestock meat	How often do you consume meat from livestock?	Everyday/Sometimes/Rarely
15		Fish and shrimp	How often do you consume fish and shrimp?	Everyday/Sometimes/Rarely
16		Eggs	How often do you consume eggs?	Everyday/Sometimes/Rarely
17		Milk and dairy products	How often do you consume milk and dairy products?	Everyday/Sometimes/Rarely
18		Vegetables	How often do you consume vegetables?	Everyday/Sometimes/Rarely
19		Fruits	How often do you consume fruits?	Everyday/Sometimes/Rarely
20		Seaweed and mushrooms	How often do you consume seaweed or mushrooms?	Everyday/Sometimes/Rarely
21		Beans	How often do you consume beans?	Everyday/Sometimes/Rarely
22		Fatty foods	How often do you consume fatty foods?	Everyday/Sometimes/Rarely
23		Salty foods	How often do you consume salty foods?	Everyday/Sometimes/Rarely

**Table 2:** Baseline characteristics of the study participants.

	Total	Category 1	Category 2	Category 3	*p*-Values			
		BMI < 22	22 ≤ BMI < 25	BMI ≥ 25	Category 1 vs. 2	Category 1 vs. 3	Category 2 vs. 3	
Number of participants	15,063	9270 [61.5%]	4519 [30.0%]	1274 [8.5%]	*p* for goodness-of-fit < 0.001			a
(men/women)	(6769/8294)	(3230/6040)	(2761/1758)	(778/496)	<0.001	<0.001	>0.999	b
Age (years)	47.1 ± 10.2	46.5 ± 10.3	48.2 ± 10.2	47.3 ± 9.5	<0.001	0.031	0.018	c
Body weight (kg)	58.1 ± 10	53.0 ± 7.1	64.3 ± 7.1	73.6 ± 9.0	<0.001	<0.001	<0.001	c
BMI (kg/m^2^)	21.4 ± 2.6	19.8 ± 1.5	23.2 ± 0.8	26.6 ± 1.7	<0.001	<0.001	<0.001	c
SBP (mmHg)	111.3 ± 15.2	108.2 ± 14.1	115.0 ± 15.2	120.8 ± 15.9	<0.001	<0.001	<0.001	c
DBP (mmHg)	70.5 ± 12.1	68.1 ± 11.2	73.6 ± 12.1	77.5 ± 12.7	<0.001	<0.001	<0.001	c
FPG (mg/dL)	88.5 ± 9.8	87.3 ± 10.0	90.1 ± 9.0	91.7 ± 10.0	<0.001	<0.001	<0.001	c
HbA1c (%)	5.40 ± 0.30	5.39 ± 0.33	5.42 ± 0.33	5.50 ± 0.30	<0.001	<0.001	0.001	c
Total cholesterol (mg/dL)	200.1 ± 33.0	197.7 ± 33.0	203.8 ± 32.4	204.6 ± 33.3	<0.001	<0.001	0.738	c
TGs (mg/dL)	82.6 ± 53.6	72.9 ± 45.6	95.3 ± 57.5	108.6 ± 71.6	<0.001	<0.001	<0.001	c
HDL-C (mg/dL)	71.0 ± 17.1	74.8 ± 17.0	65.6 ± 15.5	62.1 ± 14.9	<0.001	<0.001	<0.001	c
LDL-C (mg/dL)	120.0 ± 30.2	115.5 ± 29.5	126.8 ± 29.8	128.9 ± 29.7	<0.001	<0.001	0.056	c
AST (U/L)	19.5 ± 6.5	19.2 ± 6.3	19.8 ± 6.7	20.4 ± 7.0	<0.001	<0.001	0.023	c
ALT (U/L)	18.3 ± 9.6	16.9 ± 8.2	20.0 ± 10.3	22.8 ± 13.5	<0.001	<0.001	<0.001	c
γ-GT (U/L)	29.6 ± 32.2	25.5 ± 26.8	35.1 ± 37.7	39.7 ± 41.0	<0.001	<0.001	<0.001	c

Abbreviations: BMI, body mass index; SBP, systolic blood pressure; DBP, diastolic blood pressure; FPG, fasting plasma glucose; HbA1c, hemoglobin A1c; TGs, triglycerides; HDL-C, high-density lipoprotein cholesterol; LDL-C, low-density lipoprotein cholesterol; AST, aspartate aminotransferase; ALT, alanine aminotransferase; γ-GT, γ-glutamyltransferase. *p*-value: a, chi-square goodness-of-fit test; b, chi-square test with Bonferroni correction; c, Tukey–Kramer test. SI conversion factors: To convert FPG to mmol/L, multiply by 0.0555; HbA1c to proportion of total hemoglobin, multiply by 0.01; total cholesterol, HDL-C, and LDL-C to mmol/L, multiply by 0.0259; TGs to mmol/L, multiply by 0.0113; AST, ALT, and γ-GT to μkat/L, multiply by 0.0167.

**Table 3:** Follow-up characteristics of the study participants.

	Total	Category 1	Category 2	Category 3	*p*-Values			
		BMI < 22	22 ≤ BMI < 25	BMI ≥ 25	Category 1 vs. 2	Category 1 vs. 3	Category 2 vs. 3	
Number of participants	15,063	9270 [61.5%]	4519 [30.0%]	1274 [8.5%]	*p* for goodness-of-fit < 0.001			a
(men/women)	(6769/8294)	(3230/6040)	(2761/1758)	(778/496)	<0.001	<0.001	>0.999	b
Follow-up period (years)	4.2 [2.2–5.0]	4.5 [2.8–5.0]	4.0 [2.1–5.0]	3.5 [1.9–5.0]	<0.001	<0.001	<0.001	c
Steatosis incidence	1889 [12.5%]	551 [5.9%]	898 [19.9]	440 [34.5%]	*p* for trend < 0.001			d
Age (years)	50.8 ± 10.3	50.3 ± 10.3	51.8 ± 10.3	50.5 ± 9.6	<0.001	0.776	<0.001	c
Body weight (kg)	58.8 ± 10.4	53.8 ± 7.7	64.7 ± 7.9	73.8 ± 9.8	<0.001	<0.001	<0.001	c
BMI (kg/m^2^)	21.7 ± 2.7	20.2 ± 1.8	23.5 ± 1.4	26.8 ± 2.3	<0.001	<0.001	<0.001	c
SBP (mmHg)	116.9 ± 15.3	114.1 ± 14.5	120.9 ± 15.3	125.7 ± 15.1	<0.001	<0.001	<0.001	c
DBP (mmHg)	74.2 ± 11.7	72.2 ± 11.1	77.3 ± 11.8	80.5 ± 11.9	<0.001	<0.001	<0.001	c
FPG (mg/dL)	92.7 ± 10.4	91.5 ± 9.9	94.4 ± 10.3	95.7 ± 11.9	<0.001	<0.001	<0.001	c
HbA1c (%)	5.52 ± 0.31	5.50 ± 0.30	5.54 ± 0.32	5.57 ± 0.32	<0.001	<0.001	0.010	c
Total cholesterol (mg/dL)	208.8 ± 34.0	207.7 ± 34.3	210.4 ± 33.4	211.1 ± 33.7	<0.001	0.003	0.778	c
TGs (mg/dL)	88.1 ± 59.6	79.4 ± 53.9	99.9 ± 64.5	109.4 ± 68.2	<0.001	<0.001	<0.001	c
HDL-C (mg/dL)	71.1 ± 17.7	74.9 ± 17.7	65.7 ± 16.1	62.1 ± 15.4	<0.001	<0.001	<0.001	c
LDL-C (mg/dL)	123.8 ± 30.6	120.2 ± 30.1	129.1 ± 30.6	131.8 ± 30.5	<0.001	<0.001	0.014	c
AST (U/L)	21.1 ± 7.4	20.9 ± 6.9	21.5 ± 8.2	21.8 ± 8.0	<0.001	<0.001	0.416	c
ALT (U/L)	19.6 ± 10.9	18.1 ± 9.2	21.3 ± 11.9	24.1 ± 15.5	<0.001	<0.001	<0.001	c
γ-GT (U/L)	31.1 ± 35.9	27.2 ± 31.8	36.3 ± 39.5	41.2 ± 44.9	<0.001	<0.001	<0.001	c

Abbreviations: BMI, body mass index; SBP, systolic blood pressure; DBP, diastolic blood pressure; FPG, fasting plasma glucose; HbA1c, hemoglobin A1c; TGs, triglycerides; HDL-C, high-density lipoprotein cholesterol; LDL-C, low-density lipoprotein cholesterol; AST, aspartate aminotransferase; ALT, alanine aminotransferase; γ-GT, γ-glutamyltransferase. *p*-value: a, chi-square goodness-of-fit test; b, chi-square test with Bonferroni correction; c, Tukey–Kramer test; d, log-rank test for linearity.

## Data Availability

In accordance with the Personal Information Protection Law of Japan, individual participant data from this study cannot be made publicly available. However, data from the Takeda Hospital Medical Examination Center cohort will be made available to members of the scientific and medical community for non-commercial use upon publication, following a reasonable request via email to the corresponding author, Dr. Noriko Satoh-Asahara (email: nsatoh@kuhp.kyoto-u.ac.jp).

## Abbreviations

ALT	Alanine aminotransferase
AST	Aspartate aminotransferase
BMI	Body mass index
CVD	Cardiovascular disease
DBP	Diastolic blood pressure
FPG	Fasting plasma glucose
γ-GT	γ-glutamyltransferase
HbA1c	Hemoglobin A1c
HDL-C	High-density lipoprotein cholesterol
LDL-C	Low-density lipoprotein cholesterol
MAFLD	Metabolic dysfunction-associated fatty liver disease
NAFLD	Non-alcoholic fatty liver disease
SLD	Steatotic liver disease
SBP	Systolic blood pressure
TGs	Triglycerides
